# Safer cycling in older age (SiFAr): effects of a multi-component cycle training. a randomized controlled trial

**DOI:** 10.1186/s12877-023-03816-2

**Published:** 2023-03-07

**Authors:** Veronika Keppner, Sebastian Krumpoch, Robert Kob, Anja Rappl, Cornel C. Sieber, Ellen Freiberger, Hanna Maria Siebentritt

**Affiliations:** 1 Institute for Biomedicine of Aging, Friedrich-Alexander-Universität Erlangen-Nürnberg, Kobergerstraße 60, 90408 Nürnberg, Bavaria, Germany; 2 Department of Medical Informatics, Biometry and Epidemiology, Friedrich-Alexander-Universität Erlangen-Nürnberg, Erlangen, Bavaria, Germany; 3 Department of Medicine, Kantonsspital Winterthur, Winterthur, Switzerland

**Keywords:** Aging, e-bike, Cycling safety, Cycle training, Mobility, Randomized controlled trial

## Abstract

**Background:**

The risk of older adults being injured or killed in a bicycle accident increases significantly due to the age-related decline of physical function. Therefore, targeted interventions for older adults to improve safe cycling competence (CC) are urgently needed.

**Methods:**

The “Safer Cycling in Older Age” (SiFAr) randomized controlled trial investigated if a progressive multi-component training program related to cycling improves CC of older adults. Between June 2020 and May 2022, 127 community-dwelling persons living in the area Nürnberg-Fürth-Erlangen, Germany aged 65 years and older were recruited, who are either (1) beginners with the e-bike or (2) feeling self-reported unsteadiness when cycling or (3) uptaking cycling after a longer break. Participants were either randomized 1:1 to an intervention group (IG; cycling exercise program, 8 sessions within 3 months) or an active control group (aCG; health recommendations). The CC as primary outcome was tested not blinded in a standardized cycle course prior and after the intervention period and after 6–9 months, which consists of variant tasks requiring skills related to daily traffic situations. Regression analyses with difference of errors in the cycling course as dependent variable and group as independent variable adjusted for covariates (gender, number of errors at baseline, bicycle type, age and cycled distance) were performed.

**Results:**

96 participants (73.4 ± 5.1 years; 59.4% female) were analyzed for primary outcome. Compared to the aCG (n = 49), the IG (n = 47) made an average of 2.37 fewer errors in the cycle course after the 3 months intervention period (p = 0.004). People with more errors at baseline had higher potential for improvement (B=-0.38; p < 0.001). Women on average made 2.31 (p = 0.016) more errors than men, even after intervention. All other confounders had no significant effect on the difference in errors. The intervention effect was very stable until 6–9 months after the intervention (B=-3.07, p = 0.003), but decreased with a higher age at baseline in the adjusted model (B = 0.21, p = 0.0499).

**Conclusion:**

The SiFAr program increases cycling skills among older adults with self-perceived needs for improvement in CC and could easily be made available to a broad public due to its standardized structure and a train-the-trainer approach.

**Trial Registration:**

This study was registered with clinicaltrials.gov: NCT04362514 (27/04/2020), https://clinicaltrials.gov/ct2/show/NCT04362514.

**Supplementary Information:**

The online version contains supplementary material available at 10.1186/s12877-023-03816-2.

## Background

In Europe, the popularity of cycling as an affordable, environmentally friendly and convenient form of physical activity is reflected in increasing sales figures of bicycles, especially electrically assisted bicycles (e-bikes^1^) [1, 2]. In view of the growing number of cyclists and demographic change, it is expected that the proportion of older cyclists will also increase.

Cycling as a part of regular physical activity is related to health and functional benefits, even for older adults with chronic conditions [3, 4]. Several studies showed that cycling can improve cardiovascular, functional [5] and cognitive health parameters [6] and lower the risk of all-cause mortality [7]. In addition, regular cycling promotes independence and mobility into old age [8], which is of great individual and social importance.

However, cycling poses potential risks for older adults. The higher vulnerability caused by the age-related decline of physical and cognitive function might essentially affect the ability to avoid accidents and safe cycling behavior (e.g. reaction, coordination, motor competence). This puts older persons at a higher risk of being seriously injured in a bicycle accident, especially as e-bike riders [9, 10]. In Europe, almost half (47%) of cyclist fatalities in 2019 were cyclists aged 65 years or older [11]. In order to counteract the increasing number of serious injuries [10, 11] and to support mobility among older adults, effective training interventions are needed to improve safe cycling skills.

Previous cycling-related interventions mainly examined the effect of cycling on specific health outcomes [6, 12] or functional abilities [13, 14] among older adults. Most of these were carried out under “laboratory conditions”, i.e. on stationary bikes [5, 13], and did not reflect cycling in the real world.

To date, existing intervention studies which aimed at improving physical and cognitive skills required for safe cycling mainly targeted children [15] or adolescents [16, 17]. Hagemeister et al. [18] from the Technical University of Dresden conducted an intervention study with older adults to improve physical skills required for safe cycling, the training took place in gyms without exercises on the bicycle. Presumably because of the lack of specificity, the training program did not show significant improvement of the performance in a cycle course, which was used as the primary outcome and transfer test for safe cycling skills. Taken together, evaluated training concepts focusing the cycling competence of older adults are lacking.

### Objectives

To address this significant gap, the main objective of the “Safer Cycling on Older Age” (SiFAr) project of the Institute for Biomedicine of Aging (IBA, University of Erlangen-Nürnberg, Germany) was to test if a structured and progressive multi-component exercise program related to cycling (MEPC) for older adults improves the cycling competence (CC; e.g. balance, strength, ability to react, cycling skills and techniques). The CC was measured by completing various tasks in a cycle course in the intervention (IG) and the active control group (aCG) before and after a three month training period. The second objective was to examine if the intervention led to long-term effects on CC.

## Methods/design

### Study design and randomization

**Fig. 1:**
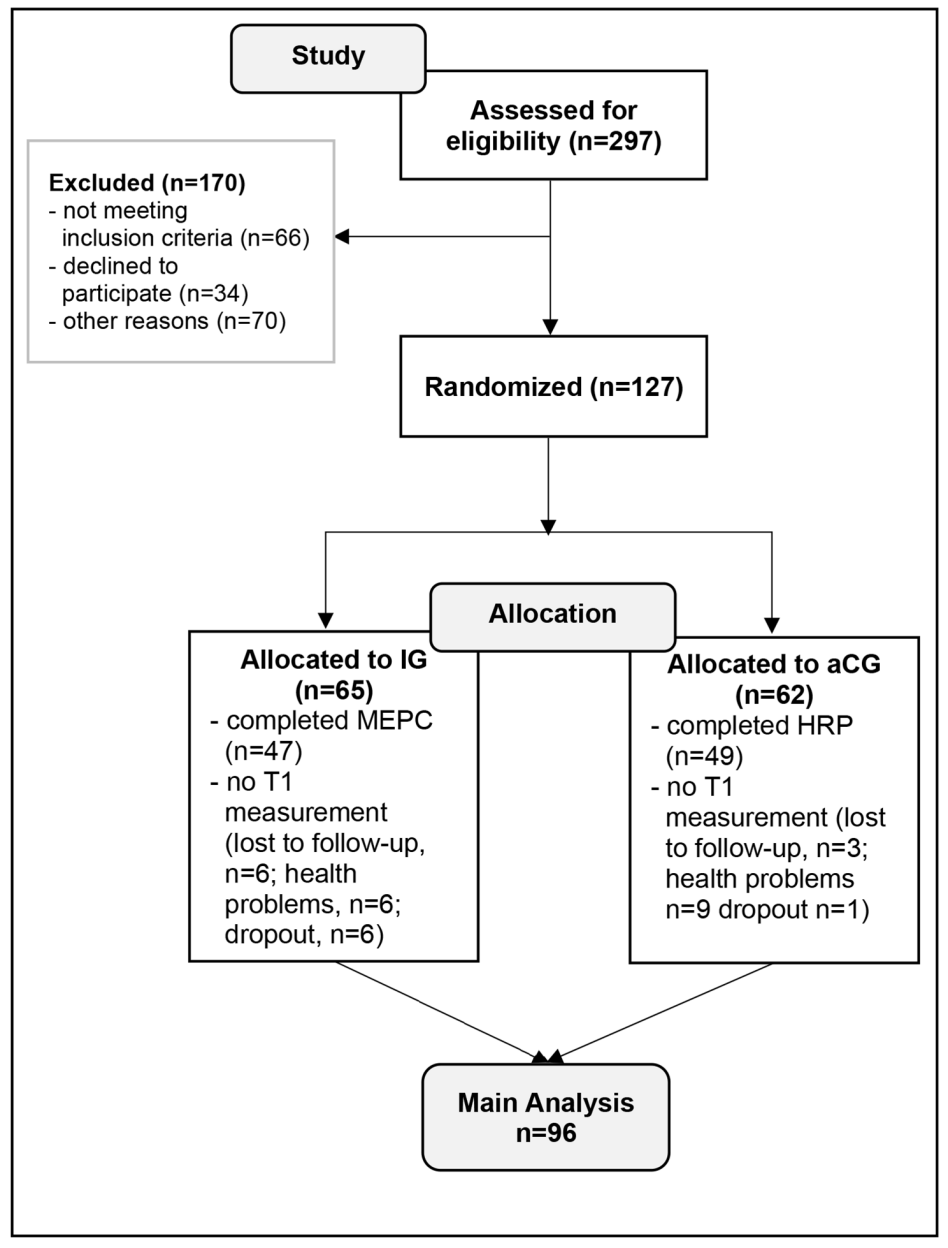
Flow Diagram

SiFAr was a parallel group, randomized controlled trial with a duration of three years and an equal allocation to intervention and control group, stratified by gender and bicycle type (e-bikes/unmotorized bicycle). Randomization of participants was performed by trained study staff after assignment of the informed consent and the baseline assessments. The randomization allocation was computer-generated via simple random sampling by a statistician who was otherwise not enrolled in study activities. Detailed description of the trail design and randomization procedure can be found in the flow chart (see Fig. 1) and the published study protocol [19]. Blinding of study personnel was not possible, as all examiners were involved in enrolling participants, collecting data, entering data into the database and scheduling participants. However, study staff was carefully trained to ensure the standardization of assessments. Furthermore, the intervention (MEPC) was instructed by external cycle trainers who were otherwise not involved in any other part of the study procedure.

Participant recruitment was undertaken between June 2020 and April 2022. Data collection was completed in August 2022.

### Ethics

The study protocol was approved by the ethic committee of the Friedrich-Alexander-Universität Erlangen-Nürnberg, Germany (FAU). The study was registered at ClinicalTrials.gov (identifier: NCT04362514; 27/04/2022) and followed the principles set out in the Helsinki declaration. Prior to the assessments at the beginning of the baseline visit, all participants received a written information sheet containing the most relevant study components and had to sign informed consent forms prior. Changes to the protocol were reported to ClinicalTrials.gov and approved by the ethical committee. During the period of study participation, all participants were provided insurance for the intervention and all assessments.

### Participants

Recruitment of study participants took place in the area Nürnberg-Fürth-Erlangen, Bavaria, Germany between June 2020 and April 2022 via advertisement in the local media (e.g. radio, newspaper, brochures) and by using a database of the IBA. The study sample consists of 127 community-dwelling persons aged 65 and older who are either (1) beginners with the e-bike or (2) feeling self-reported unsteadiness when cycling or (3) uptaking cycling after a longer break. Cyclists without self-reported limitations when cycling or with health conditions that contradict safe participation in the intervention were excluded. Due to Covid-19 restrictions, the targeted number of 200 participants based on a conservative power calculation [19] could not be reached. For detailed information on power calculation, exclusion criteria and enrollment procedure see Siebentritt et al. [19].

### Data collection

Baseline data collection included questionnaires (participants’ characteristics) and assessments (functional and psychological tests) at the IBA and measurement of the cycle course performance (see Supplementary file 1 “Cycle course tasks” and [19]). Regardless of participants’ adherence, same data were obtained after the three months intervention period (T1) and six-nine months after T1 (T2) as a follow-up. As the SiFAr project ends in December 2022, T2 could not be conducted for those who completed the intervention period by the end of summer 2022 (n = 27). Because of delayed recruitment in 2020 due to Covid-19, the number of participants (n = 25) enrolled in 2020 was too small to meaningfully assess a long-term follow-up (T3) 18–21 months after baseline. During the assessments and intervention period, adverse events (bicycle falls with injuries or complaints that could be caused by study participation) were recorded.

Data collection was finished in August 2022 and data entry was double-checked. All data is stored on the university network storage with a regular back-up.

#### Outcomes

CC was assessed in a standardized cycle course which was originally developed to test the motor competence of school children. We used a modified and validated version with seven tasks (see Supplementary file 1 “Cycle course tasks”) that is feasible and safe for older adults [18]. After each participant completed a test run, errors were recorded in a second run by trained study personnel and then cross-checked using video recordings. The tests in the cycle course took place at two different locations (Nürnberg and Erlangen), but a high degree of standardization was ensured (same structure of the cycle course, same surface). A detailed description of the test procedures can be found in the published study protocol [19].

Mean change in number of errors (IG vs. aCG) was tested as primary outcome between T0 (baseline) and T1 (after three months intervention period). In addition, we analyzed whether an intervention effect is sustainable to T2 (after six-nine months to T1).

### Intervention

#### Intervention group - multi-component exercise program related to cycling (MEPC)

The MEPC program is detailed in Supplementary file 2 “MEPC program”. It was developed to improve motor competence (balance, strength, cycling skills and techniques) and cognitive skills required for safe cycling. Participation in the three months intervention included eight outdoor sessions à 60 min. Participants took part with their own bicycles to ensure a good transfer to everyday life.

Each session had a thematic focus (e.g. braking, dismounting) and the same structure: welcome and brief health evaluation, exercises without bicycle (balance and strength), repetition and consolidation of the contents of the last session with bicycle, teaching of techniques by instructors, practice of the techniques by participants. Strength and balance exercises were performed in moderate intensity and were increased with progression. The sessions built on each other and followed principles of progression (increasing challenge and complexity) and specificity (training of various skills required for safe cycling). The MEPC program aimed to train the basic skills required for daily cycling, but not one or several tasks of the cycle course. The standardized intervention, led by trained bicycle instructors, took place outdoors on two different large traffic-calmed community places. Both places provided equal opportunities to set up exercises with aids (cones, hula hoops, lines) and to use surroundings (narrow paths, small hills).

#### Active control group - health related presentations (HRP)

Instead of the planned presentations, which were cancelled due to Covid-19, participants of the aCG received three health-related leaflets (one per month) covering the following topics: physiological changes with age, safety check of bicycle and traffic regulations. The same topics were part of the MEPC to control for possible effects of the received information on cycle course performance.

After finishing all parts of the aCG (presentations, T0, T1, T2 measurements), participants had the opportunity to attend the MEPC.

### Statistical methods

Participants’ characteristics will be presented as mean ± standard deviation or median for continuous variables. Dichotomous and categorical variables will be shown as absolute numbers and percentages. Chi-square test and, depending on whether normal distribution was present, independent t -test or Mann-Whitney-U test were used to compare groups at baseline.

Regression analyses of complete cases with difference of errors in the cycle course (absolute difference T1-T0) as dependent variable and group (IG/aCG) as dichotomous independent variable adjusted for covariates (gender, bicycle type, daily cycled distance, age) were performed to investigate the primary outcome. In addition, to account for the initial error level and the thus resulting potential for improvement, the number of errors at baseline (T0) were included in the linear model. Prior to the analyses, data were checked for normality (Q-Q-Plots, histograms) and outliers (standardized residuals, Cook’ distance).

Further, to analyze possible long-term effects of the intervention, we modeled the error differences made at T1 and T2 to baseline (T0) with help of a linear mixed model in order to account for intrasubject variation. In addition to time and group, we again included the initial error level at baseline (T0) and adjusted the model for the same potential confounders as the linear model.

IBM SPSS® Statistics for Windows, Version 26 software (IBM Corp., Armonk, NY, U.S.) and R (R-4.1.3) were used for all statistical analyses. To correct for multiple testing, Bonferroni-Holm-adjustment of p-value was applied for chi-square tests or independent t-tests/Mann-Whitney-U test (p ≤ 0.003). The level of significance for regression analysis was set at α = 0.05 and evaluated based on confidence intervals (CI).

## Results

### Participant recruitment and flow

127 persons were included in the study. Baseline measurements were completed by 118 participants and T1 assessment by 96 participants (see flow chart Fig.1). Description of participants’ characteristics and analysis of primary outcome is based on those 96 participants with complete data for T0 and T1. There were no significant differences between the complete cases sample and dropout sample in terms of age, gender and e-bike use.

As the SiFAr program ended in December 2022, T2 could only be conducted for participants enrolled in 2020 and 2021. From the originally 77 possible participants for T2, the assessment was completed by 63 participants (aCG = 35; IG = 28). Nine participants allocated to IG (lost-to-follow-up = 1, health problems = 6, dropout = 2) and five participants allocated to aCG (health problems = 5) did not complete T2 measurement.

### Participants’ characteristics

The average age of the 96 participants was 73.4 ± 5.1 years and 57 (59.4%) were women. 88.5% were included in the study because they felt self-reported unsteadiness when cycling, 5.2% had switched to an e-bike within the last year and 6.3% restarted cycling after a longer break. Table 1 provides a summary of the baseline participants’ characteristics separated by IG and aCG. No significant differences were found between groups at baseline. Overall, sum scores of measurements for health and functional characteristics describe a relatively healthy sample of older adults (see Table 1).

**Table 1:** Baseline Characteristics of Participants (n = 96)

	IG (n = 47)		aCG (n = 49)	
	n/mean	%/SD	n/mean	%/SD
Age [years]	72.5	± 4.7	74.3	± 5.3
Women	30	63.8%	27	55.1%
Living alone	20	42.6%	19	38.8%
Medications [prescribed]	3.9	± 3.0	3.5	± 2.4
Number of diseases [diagnosed]	2.1	± 1.4	2.0	± 1.7
BMI [kg/m^2^]	26.6	± 4.1	26.3	± 4.5
SPPB [score, 0–12 p.]	11.3	± 0.9	11.0	± 1.4
MoCa [score, 0–30 p.]	26.6	± 2.1	26.4	± 2.3
FES-I [score short form, 7–28 p.]	7.7	± 1.8	8.2	± 1.7
EQ-5D vas [scale, 0-100]	79.3	± 10.1	77.1	± 12.2
E-bike	24	51.1%	24	49.8%
Bicycle accident [after age of 60, yes]	26	55.3%	27	55.1%
Bicycle accident [after age of 60, number]	1.6	± 0.7	2.3	± 2.7
Single bicycle-accidents [after age of 60, yes]	18	38.3%	15	30.6%

Notes: BMI Body Mass Index, SPPB Short Physical Performance Battery, MoCA Montreal Cognitive Assessment, EQ-5D-vas EurQol-5 Dimension Visual Analogue Scale; FES-I Falls Efficacy Scale International

### Outcomes

#### Primary outcome

All participants of the IG included in the following analysis have attended at least six of the eight training sessions and were therefore considered as “adherent”. In most cases, the missed sessions could be made up by the instructors offering catch-up sessions before the regular session started.

Descriptively, mean errors in the cycle course were 9.30 ± 6.96 (T0) and 6.66 ± 4.81 (T1) in the IG versus 8.98 ± 7.80 (T0) and 8.84 ± 6.92 (T1) in the aCG (see Supplementary file 3 “Cycle course errors”). The results of the linear model (LM) with the difference in errors (T1-T0) as dependent variable are displayed in Table 2. Compared to the aCG, the IG made an average of 2.37 fewer errors in the cycle course after the three months intervention period (p = 0.004) when baseline errors were taken into account. The effect of the number of errors at baseline showed that for each error more at T0, there was an average improvement of 0.38 errors at T1 (p < 0.001). These effects remained almost unchanged when adjusted for confounding factors.

The adjusted model showed a significant gender effect. Women on average made 2.31 (p = 0.016) more errors than men. All other confounders had no significant effect on the difference in errors.

**Table 2:** Effects of the intervention after 3 months (T1, LM) and 6–9 months (T2, LMM)

	Dependent variable: difference in errors to baseline			
	linear model (LM)		linear mixed model (LMM)	
	raw model	adjusted model	raw model	adjusted model
Constant	3.30*** (1.86, 4.75)	3.76 (-8.77,16.29)	3.48*** (1.53, 5.43)	-12.77 (-28.11, 2.58)
Intervention group	-2.37** (-3.93, -0.82)	-2.51** (-4.05, -0.96)	-3.07** (-4.99, -1.16)	-3.02** (-4.91, -1.14)
Cycle course errors baseline (T0)	-0.38*** (-0.49, -0.28)	-0.45*** (-0.57, -0.32)	-0.41*** (-0.53, -0.29)	-0.50*** (-0.64, -0.36)
Women		2.31* (0.450, 4.172)		2.34** (0.13, 4.54)
E-bike		-0.44 (-2.03, 1.15)		-0.48 (-2.40, 1.44)
Cycled distance (daily average)		-0.15 (-0.41, 0.12)		0.12 (-0.15, 0.39)
Age		-0.01 (-0.17, 0.16)		0.21* (0.01, 0.42)
Time			-0.001 (-0.01, 0.01)	0.0003 (-0.01, 0.01)
Cases	96	96	63	60
Observations	96	96	123	123
Adjusted R^2^	0.38	0.42		
Akaike Inf. Crit.	538.14	535.43	693.71	694.49
Bayesian Inf. Crit.	548.40	555.86	710.38	721.94

Notes: *p < 0.05, **p < 0.01, ***p < 0.001; Inf. Crit. Information Criterium

#### Long-term-effects

Following the law of parsimony and a previously conducted sensitivity analysis a random intercepts model proved as sufficient for capturing the intrasubject variation. The results of the linear mixed model (LMM) can be found in Table 2. The intervention group made an average of 3.07 (p = 0.003) fewer errors in the cycle course over time than the control group. Time itself had no influence on the errors made (-0.001, p = 0.772). Further, the number of errors at baseline (-0.41, p < 0.0001) was significant and in line with the findings from the LM that people with more errors at baseline have higher potential for improvement. These effects were not influenced by the adjustment for confounding factors. However, the adjusted model of the LMM showed a very similar gender effect as the LM. Women on average made 2.34 (p = 0.042) more errors than men. In contrary to the LM, the LMM revealed a significant age effect. With every additional year of age of the participants at baseline on average 0.21 (p = 0.0499) significantly more errors in the cycle course were made over time. The cycled distance (daily average) had no significant influence, neither had the bicycle type.

### Adverse events

Bicycle related falls with minor injuries occurred in two participants during the activities recommended in the study. One bicycle-related fall occurred during the cycling course assessment (torn ligament) and one bicycle-related fall during the intervention (knee contusion). The latter participant was able to continue with the intervention.

## Discussion

To the best of the authors’ knowledge, this is the first study to show that a targeted cycling training program with a duration of three months improves cycling competence in older people.

Compared to the aCG, participants of the IG made an average of 2.51 less errors in the cycle course after intervention, unaffected of baseline cycle course performance, gender, bicycle type, age, and cycled distance (daily average). As the cycle course is very challenging, an improvement to a flawless performance was not expected. Descriptively, the IG improved in all skills measured in the cycle course compared to the aCG, except for riding curves (slalom) and slow cycling (see Supplementary file 3 “Cycle course errors”). Regarding the latter, there was hardly any potential for improvement, as few errors were made at baseline. For the slalom task, we assume that IG participants did not improve because they intentionally missed a cone to get safely through the cycle course or touched a cone while attempting the task.

The effect of the training program remained stable over six to nine months after the intervention, even though the long-term measurement was carried out after the winter period when people cycle less or not at all. However, it was found that a higher inclusion age of the participants weakened the long-term effect. This could be due to the fact that with increasing age, training breaks might have a greater influence on cycling competence and continuous training is indispensable (use it or lose it) [20]. Furthermore, we assume that this effect results from an interaction of the different factors of the age-related decline of function.

The effects of the SiFAr intervention were evident in a very healthy and fit collective of older people, who would not have been expected to have any limitations or great potential for improving cycle competence if only health and functional parameters were considered. Our study demonstrated that the need for bicycle training among older active people should be determined not only on the basis of physical parameters, but mainly on the basis of subjectively perceived need. Self-reported limitations seem to be a very early marker for further decline as research has shown in different areas, e.g. in fall [21] or dementia research [22].

On average, women made 2.31 more errors than men and this effect persisted over time. There could be several reasons for this finding: Women of this generation are possibly more inexperienced in cycling than men, at least as far as the period before retirement age is concerned. Men historically cycle for both leisure-oriented but also work-related reasons (commuting to work, etc.), whereas women tend to cycle only for leisure-oriented reasons [23, 24]. However, these gender differences seem to even out with retirement age [25]. Another reason could be that women more often reported to feel insecure when cycling than men [25, 26], as it was the case among our participants (40.3% vs. 6.3%). In relation to our results, this could mean that male participants were more likely to attempt a task rather than skip it due to their higher feeling of safety [27]. Women might have behaved more cautiously in the cycle course due to perceived insecurity and, for example, might have skipped a difficult task rather than taking a risk. However, this does not automatically mean that women cycle more unsafely, which is underlined by the fact that our female and male participants report the same number of bicycle falls. Nevertheless, the finding point to the importance of addressing insecurities on a psychological level within the training program and analyzing which safety strategies can be usefully implemented.

The intervention effect was independent of the bicycle type used, suggesting that the SiFAr program successfully addresses the different characteristics and challenges of both unmotorized bicycles and e-bikes. As already suspected in our study protocol [19], the “positive” and “negative” characteristics of the different types seemed to balance each other out. Some tasks such as dismounting, mounting and slow cycling [26, 28] might have been more difficult with an e-bike due to its heavier weight and speed control [29], while lane keeping and slalom seem to be easier due to the speed assistance of the e-bike [30].

Based on our observations, we assume for older adults that the individualized bicycle equipment and settings have a stronger influence on the performance in the cycle course than the bicycle type. We had the impression that dismounting and mounting was much easier for those with a low step frame than for those with a high top tube. Also a lower saddle height seemed to improve dismounting and mounting by allowing the foot to make contact with the ground sooner [31]. Furthermore, a lower handlebar height might be associated with a better balance control on the bicycle. Since only a few of the factors mentioned (e.g. saddle height) could be changed during the intervention, the effect of the SiFAr program might be greater with an optimal individualized adaptation of the bicycle settings. In addition, switching to other tbicycle types (tricycles or bicycles with small tires) might improve cycling competence and be an important factor in enabling older people with functional limitations to remain mobile. Unfortunately, not all aspects of bicycle fitting and bicycle type could be systematically recorded, but it would be highly recommended for future studies.

### Strengths and limitations

The strong acceptability of the intervention by the participants, the relatively high adherence levels and rarity of adverse events leading to injuries underline the success of the SiFar program. Participants who had many limitations in their cycling competence at baseline benefited the most. Due to the standardized structure and a train-the-trainer approach, the intervention could easily be made available to a broad public.

A further strength of the study is that the errors in the cycle course were recorded as a discrete rather than a categorical variable as in the study by Hagemeister et al. [18]. This allowed for a more accurate analysis of changes in the cycle course performance. Furthermore, the errors were documented by two independent testers during the assessment and then reviewed again by two study staff members using video recording to ensure accurate and standardized data collection. In contrast to the study by Hagemeister et al. [18], the cycle course could be performed on both test sites with the same test setup and never had to be modified.

Despite these strengths, we acknowledge some limitations. The assessment in the cycle course can only be used as a proxy for cycling competence in real life traffic behavior. However, the seven tasks of the cycle course and the SiFAr program address all basic skills required for safe cycling who can be trained on an individual level. The high proportion of single accidents among cyclists in outside built-up areas [9] supports the relevance of individual cycling competence.

Individuals who were unable to ride a bicycle due to insecurities could not be included in the study because, as participants had to come to the training sessions independently on their own bicycles. This seems to be the main limitation, that those individuals with presumably the greatest need and potential for improvement could not be included in the study. However, these people could have participated in the program if they had a bicycle customized to their needs, e.g. a tricycle.

Furthermore, it was a challenge for the study staff to motivate the participants to go to their performance limits without disregarding their safety. Therefore, there could be a difference between the actual and the theoretically possible cycling performance. Since the measurement in the cycle course took place outdoors, various influencing factors (heat, rain, leaves) cannot be prevented completely. In addition, the test person’s physical condition on the day or other factors (e.g. nervousness or stress) influence the performance in the cycle course. Since there was also a slight improvement in the control group after the intervention period, we assume that the participants were already sensitized by the measurement in the cycle course and therefore changed certain forms of cycling behavior.

## Conclusion

The SiFAr intervention was specifically designed to meet the needs of older people and leads to improvements in cycling skills in an objectively healthy sample with self-perceived needs for safer cycling competence. Participation in SiFAr thus promotes mobility and independence in older age. The training can be offered regardless of bicycle type and is still effective at older ages, although the long-term effect is slightly weakened. When switching to a different type of bicycle, participation in a bicycle-specific intervention like the SiFAr program might be beneficial to learn the handling of the changed characteristics. The standardized structure and the train-the-trainer approach enable a fast and broad implementation in community-settings. Further studies are needed to analyze the influence of different bicycle characteristics on the cycling competence of older adults.

## Electronic supplementary material

Below is the link to the electronic supplementary material.

Supplementary Material 1. Cycle course tasks

Supplementary Material 2. MEPC program

Supplementary Material 3. Cycle course errors

## Data Availability

The datasets used and/or analysed during the study are available from the corresponding author on reasonable request.

## Footnotes

1 For this paper the term e-bike will be used exclusively to refer to electrically-assisted bicycles which require the rider to pedal and with pedal assistance up to 25 km/h.

## Abbreviations

aCG: Active control group
BMI: Body mass index
CC: Cycling competence
CI: Confidence intervals
EQ-5D-vas: EurQol-5 Dimension Visual Analogue Scale
FAU: Friedrich-Alexander-Universität Erlangen-Nürnberg, Germany
FES-I: Falls Efficacy Scale- International Version
HRP: Health related presentations
IBA: Institute for Biomedicine of Aging
IG: IG
LM: Linear model
LMM: Linear mixed model
MEPC: Multi-component exercise related to cycling
MoCA: Montreal- Cognitive Assessment
SiFAr: Safer Cycling in Older Age
SPPB: Short Physical Performance Battery
