# Supplementary material for: Safer cycling in older age (SiFAr): effects of a multi-component cycle training. a randomized controlled trial
Source: BMC Geriatr. 2023 Mar 7;23:131. doi: 10.1186/s12877-023-03816-2 (PMC9990551; doi:10.1186/s12877-023-03816-2)
Supplement: Supplementary file 1 — Supplementary Material 1. Cycle course tasks [file 12877_2023_3816_MOESM1_ESM.docx]

**Supplementary file 1** Cycle course tasks

| **Task** | **Instruction** | **Rating – yes/no and number of errors** |
| --- | --- | --- |
| **1. Slalom** | - *entrance from the right side* - *driving through all 7 cones in Slalom-style without touching the cones or the ground* | - wrong direction of entering parcours - touching or missing a cone - pushing with foot or touching the ground - passage in standing position |
| **2. Slow cycling** | - *passage of the corridor as slowly as possible without touching the marked sidelines or the ground* | - touching a line with the front wheel - touching the ground - passage in standing position - riding too fast (< 5 seconds) |
| **3.a Dismounting to both sides into a hula hoop** (right side first) | - *accurate stopping next to the hula hoop* - *both feet must be placed into the hula hoop one after another without touching the ring (leg proximal to the hula hoop must go first)* | - missing or touching the hula hoop - dismounting on wrong side - entering the hula hoop with only one leg before getting on the bicycle - foot not placed directly from the pedal into the hula hoop - tipping over of the bike |
| **3.b Mounting the bicycle out of the hula hoop from both sides**  and initiate to ride | - *leg closer to the bicycle must be moved directly from the hula hoop onto the pedal* - *initiate riding with simultaneously placement of second leg on pedal* | - failing to mount bicycle from a standing position - foot not placed directly from the hula hoop onto the pedal |
| **4. Narrow alley** | - *passage of the corridor without touching the marked lines* | - touching the ground - touching the line with the front wheel |
| **5. Turning to the left side** | - *riding straight inside the marked corridor* - *initiation of the turning process by well-timed hand-sign and look over the shoulder* - *capture of the shown number by looking behind* - *turning left within the curved corridor* | - missed look over the shoulder - missed arm signal/hand-sign - missed or erroneous naming of the number - touching the lines with the front-wheel - touching the ground |
| **6. Precise braking** | - *cycling straight at increased speed* - *precise braking within the corridor (ground-front wheel contact point within the corridor)* | - stopping before or behind the corridor - back wheel fish-tailing - jumping off the bicycle or braking with the feet - slow speed before stopping |


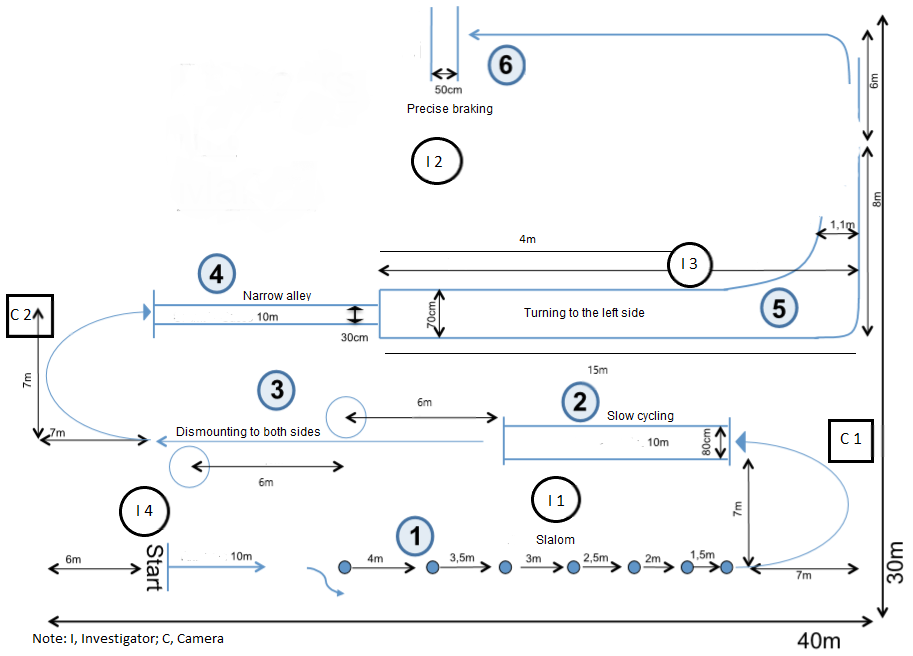


**Figure 2** Cycle course (adapted from Hagemeister & Bunte, 2014); I, Investigator; C, Camera
