# Supplementary material for: Safer cycling in older age (SiFAr): effects of a multi-component cycle training. a randomized controlled trial
Source: BMC Geriatr. 2023 Mar 7;23:131. doi: 10.1186/s12877-023-03816-2 (PMC9990551; doi:10.1186/s12877-023-03816-2)
Supplement: Supplementary file 2 — Supplementary Material 2. MEPC program [file 12877_2023_3816_MOESM2_ESM.docx]

**Supplementary file 2** MEPC program

| **Session 1:** Mounting/Dismounting the bicycle |
| --- |
| - short introduction of the cycling course - bike check (road safety and ergonomics) - introduction of balance and strength exercises (Semi-Tandem; wide leg squats, 2x10 repetitions) - information on general behavior and safety instructions in the cycling course - exercises for getting on and off the bicycle on both sides |
| **Session 2:** Braking |
| - balance exercises (variations of Semi-Tandem-stand, Tandem-stand and one leg-stand with 2x10 s holding time per side) and strength exercises (wide leg squats, hip flection, 2x 10 repetitions^1^) - consolidation of previous session: getting on and off the bicycle at marked position or on command - exercises for precise braking from different speeds - teaching of braking techniques and environmental influences on braking distance |
| **Session 3:** Riding curves |
| - balance and strength exercises - consolidation of previous session: braking/getting of the bicycle to various commands, discussing the effects of divided attention - teaching and practicing of curve techniques (different speeds and changing curve radius, avoiding obstacles) |
| **Session 4:** Track-keeping |
| - balance and strength exercises - consolidation of previous session: riding curves one-handed, ride along a partner - teaching and practicing of techniques to stay on the track: riding through a narrow corridor at various speeds, transfer to real-life situations |
| **Session 5:** Turning to the left |
| - balance and strength exercises - consolidation of previous session: skill exercises for track-keeping (partner exercises, „snail race“) - teaching and practicing of techniques for turning to the left (hand-sign, looking over the shoulder) while riding in a straight line |
| **Session 6:** Turning to the left |
| - balance and strength exercises - consolidation of previous session: riding one-handed (hand-sign) and look over each shoulder while riding in a straight line - practicing techniques for the 8 steps of turning left in line with traffic regulations |
| **Session 7:** Divided attention/complex situations |
| - balance and strength exercises - practicing cycling with additional cognitive tasks (calculation, conversation with partner and memorizing the contents of the conversation) and in complex situations (cycling an 8) - teaching of cycling-related traffic regulations |
| **Session 8:** Skills exercises/Practicing |
| - balance and strength exercises - teaching of safe traffic behavior (e.g. blind spot, anticipation of mistakes by other drivers) - Skill exercises for integration of practiced driving techniques (e.g. “shadow riding” with partner) - course reflection: exchange of experiences |

^1^since each contains a variation of these balance and strength exercises, these are no longer described in detail
