# Supplementary material for: Safer cycling in older age (SiFAr): effects of a multi-component cycle training. a randomized controlled trial
Source: BMC Geriatr. 2023 Mar 7;23:131. doi: 10.1186/s12877-023-03816-2 (PMC9990551; doi:10.1186/s12877-023-03816-2)
Supplement: Supplementary file 3 — Supplementary Material 3. Cycle course errors [file 12877_2023_3816_MOESM3_ESM.docx]

**Supplementary file 3** Cycle course errors

|  | **IG**  **(*n* = 47)** | | | | | | **aCG**  **(*n* = 49)** | | | | | |
| --- | --- | --- | --- | --- | --- | --- | --- | --- | --- | --- | --- | --- |
|  | **T0** | | **T1** | | **T2^1^** | | **T0** | | **T1** | | **T2^2^** | |
|  | **M** | **SD** | **M** | **SD** | **M** | **SD** | **M** | **SD** | **M** | **SD** | **M** | **SD** |
| Cycle course errors (total) | 9.30 | ±6.96 | 6.66 | ±4.81 | 5.13 | ±4.37 | 8.98 | ±7.80 | 8.84 | ±6.92 | 9.38 | ±7.67 |
| Slalom | 1.40 | ±1.33 | 1.55 | ±1.32 | 0.92 | ±0.93 | 1.51 | ±1.49 | 1.45 | ±1.39 | 1.78 | ±1.84 |
| Slow cycling | 0.23 | ±0.43 | 0.21 | ±0.55 | 0.21 | ±0.41 | 0.45 | ±0.82 | 0.41 | ±0.79 | 0.28 | ±0.52 |
| (Dis)mounting right side | 2.85 | ±2.87 | 2.13 | ±2.31 | 1.42 | ±1.72 | 2.47 | ±2.96 | 2.47 | ±2.67 | 2.41 | ±3.12 |
| (Dis)mounting left side | 2.94 | ±3.05 | 1.89 | ±2.11 | 1.67 | ±2.06 | 2.70 | ±2.67 | 2.84 | ±2.98 | 2.97 | ±3.44 |
| Narrow alley | 0.57 | ±0.80 | 0.32 | ±0.56 | 0.50 | ±0.66 | 0.60 | ±1.19 | 0.43 | ±0.76 | 0.47 | ±0.76 |
| Turning to the left side | 1.00 | ±1.38 | 0.50 | ±0.69 | 0.25 | ±0.44 | 1.00 | ±1.53 | 0.92 | ±1.08 | 1.19 | ±1.53 |
| Precise braking | 0.30 | ±0.55 | 0.13 | ±0.40 | 0.17 | ±0.38 | 0.29 | ±0.58 | 0.33 | ±0.55 | 0.79 | ±1.28 |

Notes: M mean value, SD standard deviation, ^1^n=24; ^2^n=32
